# Book Reviews

**Published:** 1903-10

**Authors:** 


### BOOK REVIEWS.

THE DIAGNOSIS OF DISEASES OF WOMEN. A Treatise for Students and Practitioners. By Palmer Findley, M. D., Instructor in Obstetrics and Gynecology in Rush Medical College, in affiliation with the University of Chicago. In one octavo volume of 494 pages, illustrated with 210 engravings and 45 full-page plates in colors and monochrome. Philadelphia and New York: Lea Brothers & Co. 1903. (Cloth, $4.50 net; leather, $5.50.)

This treatise will be found of great use to the student of pathology, whether he be undergraduate or practitioner. In this it follows the lines of the German books that deal so extensively with morbid anatomy. Many of our gynecological treatises, excellent though they be, are somewhat deficient in this respect. Findlay's work will supplement them most admirably.

The author has not failed to give due attention to the clinical aspects of his subject, which is a desideratum for both the student and practitioner, to whom the book is particularly commended, these composing the audience for which it was written. Whoever labors under the delusion that gynecology as a specialty is passing, should read the book with care. Every page bristles with the importance of special knowledge for the conduct of the diseases of women. Those who would succeed must be familiar with the use of the microscope, for many affections cannot be given their appropriate place without the aid of high-power lenses.

The illustrations deserve a word of praise, many of which are microscopic sections of diseased conditions observed by the author or others, and all of which are helpful to a better understanding of the text. If another work is to be added to the already massive literature of gynecology it is well that it is constructed on the lines laid out by Findley. He will be sure of a numerous and attentive audience that cannot fail to be instructed as well as interested by what he has written.

A REFERENCE HANDBOOK OF THE MEDICAL SCIENCES BY VARIOUS WRITERS. New edition. Edited by ALBERT H. BUCK, M. D. Volume VI. Imp. Quarto. 1012 pages. 764 engravings. Nine full-page plates, in black and colors. New York: William Wood & Company. 1903.

Whoever is sceptical as to the usefulness of such a series as is being issued under the title of Reference Handbook of the Medical Sciences, should examine with care the sixth volume which is recently from the press. It contains over a thousand pages of important, interesting, and instructive scientific material, by authors of acknowledged ability, the great majority of whom are entitled to speak *ex cathedra* on the subjects of which they write.

We may not be invidious where all is of so much value, yet we would invite special attention to the article by Professor Vander Veer, of Albany, on retroperitoneal tumors, which is a succinct setting forth of our present knowledge on this class of new growths. It exhibits a clear understanding of the pathology, histology, and treatment of these neoplasms,—there being but one life-saving method of the latter, and that surgical. Dr. Vander Veer's article in a former volume on cleft palate, was a masterful handling of a technical subject, characterised by the same skilful knowledge as marks the present monograph. We may mention, however, the articles on muscles, by Prof. Simon H. Gage, Lydia M. Dewitt and Francis J. Shephard; on nasal cavities, by Robert C. Myles; on parasites, by Henry B. Ward; on the nervous system, by Pearce Bailey, James J. Putnam, Wm. M. Leszynsky and Lewellys F. Barker; on ophthalmological topics, by John Green and Beaumont Small; on ovariotomy, by Hunter Robb; on the ovum, by Robert P. Bigelow; on the pelvis, by Frank Baker; on the pharynx, by Seth Scott Bishop; on pleurisy, by Frank W. Jackson; on pneumonia, by Andrew H. Smith; on poisonous plants, by Henry H. Bushby; on poisonous reptiles, by Gustav Langmann; on poisons, by Charles Harrington and R. H. C. Chittenden; on the prostate, by Arthur T. and Hugh Cabot; on ptomains, by Rudolph A. Witthaus; on the pulse, by Wm. S. Morrow; on reflexes, by Joseph Fræenkel; on reparative surgery, by Joseph Ransohoff; on resection of joints, by Frank Hartley, and on the Röntgen rays, by Wm. C. Borden. This volume contains 764 engravings and nine insert plates in black and colors.

---

TUBERCULOSIS. Recast from Lectures Delivered at Rush Medical College, Chicago. By NORMAN BRIDGE, A. M., M. D., Emeritus Professor of Medicine in Rush Medical College. Philadelphia, New York, London: W. B. Saunders & Company. 1903. (Cloth, $1.50 net.)

The disease of which this modest volume treats has become the scourge of the human race, at least of the white portion of it, for, as declared by this author, tuberculosis in one form or another, is responsible for the destruction of one-ninth of all the white races. Surely, such destructiveness of life is worthy the name of scourge; and again, quite as surely it is worthy the best efforts of the medical profession to check, if not arrest its spread through channels of infection.

Since Koch, about 20 years ago, pointed out its microbic origin, activity has been manifest all over the civilised world in the direction of preventing or controlling the spread of tuberculous disease, but the people are slow to adopt even the simplest measures of prevention recommended by sanitarians. If all educated persons, not physicians, could read this book, it would prove a mighty aid to the cause of prevention. Bridge has made a contribution to the study of tuberculosis in this work, and it deserves a wide-spread circulation. It is by far the most practical monograph on the subject that has yet appeared, and belongs on the book-table of every person interested in the prevention or cure of the disease with which it deals.

Lea's Series of Pocket Textbooks. A Manual of Bacteriology for Students and Physicians. By Fred C. Zapffe, M. D., Professor of Pathology and Bacteriology in the Illinois Medical College; Professor of Histology in the Department of Medicine and in the School of Dentistry of the University of Illinois, Chicago. Duodecimo, 350 pages, with 150 engravings and 7 full-page colored plates. Series edited by Bern B. Gallaudet, M. D., Demonstrator of Anatomy and Instructor in Surgery, College of Physicians and Surgeons, Columbia University, New York. Lea Brothers & Co., Philadelphia and New York. 1903. (Price, cloth, $1.50 net; flexible leather, $2.00 net.)

In order to acquire a knowledge of bacteriology,—a working clinical knowledge even,—the laboratory must be resorted to and that under an experienced instructor. But one must also have books which likewise have been prepared by a competent teacher of bacteriology. This book is just such an one; it is succinct in expression, compact in form, practical in character, and handsome in make-up. It begins at the foundation of the subject upon which it constructs a superstructure that includes the whole fabric of the science. It is distinctly a book for the student and practitioner, for the beginner and for the clinician. It is set with engravings all through the text that are gems, many of them of the first water, and which contribute vastly to a completer understanding of the author's meaning. It is by and large a most satisfactory manual, and will not disappoint the purchaser.

---

A Textbook of Minor Surgery, Including Bandaging. By Newman T. B. Nobles, M. D., Professor of Surgery at the Cleveland Homeopathic Medical College. 325 pages. Boericke & Tafel, Philadelphia. 1903. (Cloth, $2.50; postage, 15 cents).

If another work were needed on the subjects embraced by the above title perhaps this book might be regarded as filling a place: but it is difficult to appreciate any such necessity in view of the excellent manuals of Wharton and others. Moreover, this "textbook" does not deserve such a name, nor does it rise to the standard already set by similar treatises now in the field. In the bandaging part it is decidedly weak, incomplete, and altogether unsatisfactory. Nevertheless, in spite of its defects, the book contains much that is useful to the novice and may serve its purpose to the audience for which it is intended. How in the world any surgeon today, no matter in what "school" of medicine taught, could recommend belladonna, calcarea carb, hepar sulphur, mercurius, etc., in chronic abscess surpasses our comprehension.

---

A Textbook of Modern Materia Medica and Therapeutics. By A. A. Stevens, A. M., M. D., Lecturer on Physical Diagnosis in the University of Pennsylvania; Physician to the Episcopal and St. Agnes Hospitals, Philadelphia. Third edition, greatly enlarged, rewritten, and reset. Octavo of 663 pages. W. B. Saunders & Company. 1903. (Cloth, $3.50 net).

This edition represents practically a new book, as the work has been entirely rewritten. This is well for advances have been rapid and changes many since the previous edition was put forth. Drugs are now classed according to their pharmacological action, instead of alphabetically as heretofore. Old drugs have been elaborately considered and all modern knowledge concerning them has been utilised; newer drugs, that is, such as are possessed of merit, have been carefully analysed; references have been omitted in order to economise space,—these and other features of the book will at once commend themselves to the searcher for facts.

The status of Stevens as a teacher is so fully established on a high plane that whatever he has to say commands respect and carries weight, hence there is safety in accepting this textbook as a guide for the student and practitioner. It is a complete modern treatise on the subjects with which it deals.

---

The Practical Medicine Series of Year Books. Ten volumes. Issued under the general editorial charge of Gustavus P. Head, M. D., Professor of Laryngology and Rhinology in the Chicago Post-Graduate Medical School. Vol. V., Obstetrics. Edited by Reuben Peterson, M. D., Professor of Obstetrics and Gynecology, University of Michigan. 12mo, pp. 204. Chicago: The Year Book Publishers. 1903. (Price, $1.25; entire series, $7.50.)

The object of this book is to give a resume of the best obstetric literature of the year,—a purpose in which it succeeds. It is constructed on the same lines as the previous volumes by the same author. The object of the series is primarily to place in the hands of the general practitioner of medicine that for which he would search the periodical and other literature, perhaps vainly; and place it promptly without loss of valuable time. This is particularly important for the obstetrician who may need suggestive aid in a pending case, and he will here find the latest thought on pregnancy and its complications; labor and its complications; the puerperium and obstetric surgery.

We commend the book for its careful preparation of the material it contains and for its value as an aid to the busy physician who engages in the practice of obstetrics.

TWENTY-SECOND ANNUAL REPORT OF THE STATE DEPARTMENT OF HEALTH OF NEW YORK, for the year ending December 31, 1901. With maps. J. B. Lyon Company, Albany, State Printers. 1902.

This is the first report issued under the present organisation of the state health department, or since it became a single-headed commission. It contains the usual material that pertains to such documents and is arranged under the following heads: (1) the sanitary condition of the state; (2) water supplies and drainage; (3) investigation of alleged nuisances; (4) investigations ordered by the governor; (5) bureau of chemistry; (6) bureau of pathology and bacteriology; (7) bureau of vital statistics; (8) antitoxin laboratory; (9) cancer laboratory; (10) conference of sanitary officers; (11) examination of embalmers; (12) Barren Island and Cheektowago; (13) recommendations; (14) financial report.

A considerable part of the report relates to a comprehensive system of sewerage for the village of Plattsburg, which appears to have been a much needed improvement. A large collection of maps prepared by engineers accompanies the report. Several of these relate to the village of Depew. The document does not differ in appearance from those that have preceded it.

---

TRANSACTIONS OF THE AMERICAN ASSOCIATION OF OBSTETRICIANS AND GYNECOLOGISTS. WILLIAM WARREN POTTER, M. D., Secretary. Vol. XV. For the year 1902. New York: Stettiner Brothers. 1903.

The volume of annual transactions, published by this association, is equal in general appearance and quality of contents to any similar publication with which we are familiar. The meeting, the proceedings of which this volume records was held in the city of Washington, under the presidency of Edwin Ricketts, of Cincinnati. General George M. Sternberg, Surgeon-General United States Army (retired), delivered the address of welcome, which abounded in felicitous rhetoric, and was a model of good taste. The president appropriately responded, and then presented a gavel to the association, the head of which was made of a girder from the home of Ephraim McDowell, at Danville, Ky., where the first ovariotomy was performed, and the handle from a hickory joist, taken from a house near Greenfield, O., in which Alexander Dunlap, September 17, 1843, performed his first ovariotomy. The incident was one of interest and the president was accorded hearty applause and congratulation, with a vote of thanks for the gift. The scientific papers and discussions are among the best the association has yet published.

